# Study on Carbohydrate Metabolism in Adult Zebrafish (*Danio rerio*)

**DOI:** 10.1155/2023/1397508

**Published:** 2023-10-20

**Authors:** Longwei Xi, Qisheng Lu, Yulong Liu, Yulong Gong, Haokun Liu, Junyan Jin, Zhimin Zhang, Yunxia Yang, Xiaoming Zhu, Dong Han, Shouqi Xie

**Affiliations:** ^1^State Key Laboratory of Freshwater Ecology and Biotechnology, Institute of Hydrobiology, Chinese Academy of Sciences, Wuhan 430072, China; ^2^University of Chinese Academy of Sciences, Beijing 100049, China; ^3^Hubei Hongshan Laboratory, Wuhan 430070, China; ^4^The Innovative Academy of Seed Design, Chinese Academy of Sciences, Beijing 100101, China

## Abstract

Excessive carbohydrate intake leads to metabolic disorders in fish. However, few literatures have reported the appropriate carbohydrate level for zebrafish, and the metabolic response to dietary carbohydrate remains largely unknown in zebrafish. This study assessed the responses of zebrafish and zebrafish liver cell line (ZFL) to different carbohydrate levels. In vivo results showed that ≥30% dietary dextrin levels significantly increased the plasma glucose content, activated the expression of hepatic glycolysis-related genes, and inhibited the expression of hepatic gluconeogenesis-related genes in zebrafish. Oil red O staining, triglyceride content, and Hematoxylin-Eosin staining results showed that dietary dextrin levels of ≥30% significantly increased lipid accumulation and liver damage, as well as processes related to glycolipid metabolism and inflammation in zebrafish. In ZFL, the transcription factor sterol regulatory element binding protein-1c signal intensity, 4,4-difluoro-1,3,5,7,8-pentamethyl-4-bora-3a,4a-diaza-s-indacene (BODIPY 493/503) signal intensity, and triglyceride content were also significantly increased when incubated in high glucose, along with abnormal glycolipid metabolism and increased inflammation-related genes. In conclusion, we demonstrated that the maximum dietary carbohydrate level in adult zebrafish should be less than 30%. Excess dietary carbohydrates (30%–50%) caused hepatic steatosis and damage to zebrafish, similar to that seen in aquaculture species. Thus, this study assessed responses to different carbohydrate levels in zebrafish and illustrated that zebrafish is an optimal model for investigating glucose metabolism in some aquatic animals.

## 1. Introduction

Carbohydrate is one of the major dietary macronutrients, which is usually broken down preferentially when supplying energy to the body [1]. As the cheapest energy-supplying nutrient, appropriate dietary carbohydrate not only promotes fish growth performance but also effectively reduces ammonia excretion and aquaculture costs [2–4]. However, fish have a very limited ability to utilize carbohydrates, and long-term consumption of excessive carbohydrates may lead to negative effects such as growth retardation, fat accumulation, and abnormal nutrient metabolism [2, 4, 5]. Even though glucose metabolism in fish has been studied for many years, fish respond differently to carbohydrate deficiency or excess [6–8]. Therefore, there are potential development prospects for improving carbohydrate utilization in fish [3]. But a key prerequisite for studying the mechanisms of glucose metabolism is the lack of a fish model.

Zebrafish is a widely used model organism and an experimental fish with a short breeding cycle, available year-round [9–11] and considerable potential for nutrition research [12]. To date, numerous studies have been conducted on nutrient metabolism in zebrafish. For example, dietary additives such as nucleotides [13] and metallic elements [14] have been reported to influence the nutritional metabolism of fish. Furthermore, gene editing technologies—such as the CRISPR-Cas9 method—have been used to investigate the functions of key genes in the fish of protein or lipid metabolism, with the zebrafish model being a popular choice [15, 16]. Previous studies have found that the macronutrient content of zebrafish diet has been shown to affect the postprandial expression of metabolic factors [17, 18]. However, there are still gaps in our knowledge of the species' nutritional requirements, which limits its use in nutrition research. Therefore, it is essential to identify a suitable dietary carbohydrate for zebrafish glucose metabolism.

Although dietary protein and lipid requirements in zebrafish have been suggested at 37.6% to 44.8% and 8%, respectively [19, 20], no literature has yet reported carbohydrate requirements in zebrafish. This greatly limits the study of nutrient metabolism and regulation in zebrafish models, because deficient or excessive dietary carbohydrates can significantly affect the nutrient metabolism of fish. Excess dietary carbohydrate leads to over-accumulation of lipid and liver damage [21–23]. In this study, we first evaluated the effects of dietary carbohydrates on zebrafish, characterized by liver health and glucose metabolism both in vivo and in vitro. And reference dietary carbohydrate levels for studying glucose metabolism in zebrafish will be provided, including appropriate and excessive carbohydrate levels for zebrafish models.

## 2. Materials and Methods

### 2.1. Ethics Statement

Experimental zebrafish were obtained from the Institute of Hydrobiology, Chinese Academy of Sciences (Wuhan, Hubei, China). Animal experiments and treatments were performed according to the Guide for Animal Care and Use Committee of the Institute of Hydrobiology, Chinese Academy of Sciences (IHB, CAS, Protocol No. 2016-018).

### 2.2. In Vivo Study

#### 2.2.1. Experimental Diets

Dextrin was used as the main carbohydrate source in experimental diets. Six isonitrogenic and isolipid diets were formulated with dextrin content of 0% (CO), 10% (C10), 20% (C20), 30% (C30), 40% (C40), and 50% (C50) (Table 1). According to the amino acid profile of the dorsal muscle of zebrafish [24], three L-amino acids were supplemented to diets. First, all ingredients are passed through a 100-mesh screen. Second, they were completely mixed with passing through a twin-screwed extruder (Jinan Dingrun Machinery Co., Ltd., Jinan, Shandong, China). Third, all pellets were dried in an oven at 65°C. Fourth, the dried pellets are crushed by a pulverizer and screened through a 40- to 80-mesh screen, and then get the experimental diets. And the diets were stored in the −20°C refrigerator to prevent oxidation until the experiment finished. The proximate composition of diets was determined using AOAC methods [25]. Briefly, moisture content was determined by drying the samples to a constant weight at 105°C for 24 hr and calculated as the percentage of water loss. Crude protein content (*N* × 6.25) was determined after acid digestion using an auto Kjeldahl system (Kjeltec-8400, FOSS Tecator, Haganas, Sweden). Crude lipid content was determined by ether extraction in a Soxtec system (Soxtec System HT6, Tecator, Haganas, Sweden). Crude ash content was determined by incineration in a muffle furnace at 550°C for 12 hr. Fiber was measured using an enzymatic gravimetric method. Gross energy was determined using a Philips Microbomb Calorimeter (Gentry Instruments Inc., Aiken, USA). The proximate composition of the diets is shown in Table 1.

#### 2.2.2. Fish and Feeding Experiment

Eight pairs of male and female wild-type zebrafish (6 months old) from the same parent were selected as parent fish to ensure that the genetic background of the experimental fish is similar. Embryos were obtained by natural fertilization from them and were incubated in hatching water (4 L water + 6 mL sea salt + 200 *μ*L methylene blue saturated solution) with 50 embryos per dish. After 4 dpf (days post fertilization), all larvae were transferred to standing water aquariums (50 larvae/aquarium/L) and fed with milled yolk water. At 10 dpf, they were fed milled yolk water and a small amount of newly hatched brine shrimp (*Artemia cysts*) (Tianjin Fengnian Aquaculture Co., Ltd., Tianjin, China) until 15 dpf. From embryos to 15 dpf larvae, half of the rearing water was recruited daily. After 15 dpf, all larvae (60 fish/aquarium/10 L) were transferred to a circulating water system and fed with newly hatched brine shrimp twice daily until the experiment began.

The experiment was carried out in a recirculating water system containing 18 plastic aquariums (10 L/aquarium). Studies have been published on sex-specific responses to dietary macronutrients [26]. In order to avoid problems associated with metabolic differences in the two sexes, here, a total of 324 male zebrafish (100 dpf) with similar size were randomly stocked in 18 aquariums (18 fish/aquarium) after 24 hr fasting. A dose (0.085 g/L systemic water) of MS-222 (Cat. no. A5040, Sigma, St. Louis, MO, USA) was used to measure initial weight and length. Each diet was randomly assigned to three aquariums (54 fish/treatment). The experimental fish were then fed diets of 6% body weight at 09:00 and 17:00 daily for 6 weeks. During the experiment, the water temperature was kept at 28°C.

#### 2.2.3. Sampling

Fish were anesthetized using MS-222 and weight and length were measured after 24 hr fasting.

Fish were anesthetized using MS-222, and then body weight and body length were measured after 24 hr fasting. After anesthetized, six fish from each aquarium were sacrificed (by cutting the caudal fin with scissors) and their whole blood in the wound was immediately collected by heparin-treated tips, then mixed blood was centrifuged at 3,000 × *g* (4°C) for 10 min to acquire plasma, which was stored at −80°C for further analysis. After blood collection, part of the liver tissue was sampled immediately for total RNA extraction, and the other was fixed with 4% paraformaldehyde for histological analysis.

### 2.3. In Vitro Study

#### 2.3.1. Culturing of ZFL

Zebrafish liver cell line (ZFL) was kindly provided by the laboratory of Prof. Jian-Fang Gui (Institute of Hydrobiology, Chinese Academy of Sciences, China). The cells were cultured in the F-12 basic medium (glucose content: 10 mM, C11765500BT, GIBCO, Made in China, USA) supplemented with 10% certified fetal bovine serum (C04001-500, VivaCell, China) and 1% penicillin–streptomycin solution (03-031-1B, VivaCell, China) at 28.5°C in 5% CO_2_.

#### 2.3.2. High Glucose Treatment of ZFL

ZFL (about 1 × 10^5^ cells/cm^2^) were incubated in 6-well or 12-well plates (F-12 basic medium supplemented with 10% fetal bovine serum and 1% penicillin–streptomycin solution) for 24 hr, followed by the incubation under the control group (F-12 basic medium) or high glucose group (20 mM glucose in F-12 basic medium) for another 24 hr.

### 2.4. Sample Analysis

#### 2.4.1. Biochemical Assays

Plasma glucose was measured using a LabAssay Glucose Kit (298-65701, Wako Pure Chemical Industries, Tokyo, Japan). Plasma cholesterol was measured using commercially available kits (A111-1, Nanjing Jiancheng Bioengineering Institute, China). Triglyceride was measured using commercially available kits (A110-1, Nanjing Jiancheng Bioengineering Institute, China).

#### 2.4.2. Histochemical and Histological Analysis

Liver tissues were dehydrated in a graded ethanol series after 24 hr at 4% paraformaldehyde. They were then embedded in paraffin and cut into 4 *μ*m sections that were stained with histochemical (Oil red O staining) and histological (H&E staining). Oil red O stain sections were photographed (Nikon Eclipse CI, Japan) and then quantified according to their red area. Hematoxylin-Eosin (H&E) staining sections were photographed (Nikon Eclipse CI, Japan) and scored (1–2 = severe damage, 3–4 = moderate, 5–6 = mild, 7–8 = slight, and 9–10 = normal) according to the method described in our previous study [27].

#### 2.4.3. BODIPY 493/503 Staining

Treated ZFL were further incubated with 1 *μ*g/mL BODIPY 493/505 (D3922, Invitrogen) in control or high glucose medium for 8 min at room temperature to localize the lipid content of the lipid droplets. Then, the images were cleaned with phosphate buffer saline (PBS) three times, fixed at 4% formaldehyde for 40 min, cleaned with PBS for another three times, and finally, the images were photographed using confocal microscopy (SP8 DLS, Leica, Germany), analyzed by Imaris Viewer (Oxford Instruments, UK), and quantized their mean fluorescence intensity by Image J (National Institutes of Health, USA). All the above operations were under dim light.

#### 2.4.4. Immunofluorescent Staining

Immunofluorescence analysis was mainly conducted in the following steps: ZFL were fixed in 4% paraformaldehyde for 30 min, permeabilizated in 0.3% Trition X-100 (with PBS) for 5 min, blocked in 5% goat serum (with PBS) for 2 hr at room temperature and incubated by using the antisterol regulatory element binding protein-1-c (SREBP-1c; Abcam, ab28481, Rabbit) primary antibody (primary antibody : antibody dilution buffer = 1 : 200) overnight at 4°C. The following day, cells were incubated using Alexa Fluor 488 (Cell Signaling Technology, 4412, Rabbit) secondary antibody (secondary antibody : antibody dilution buffer = 1 : 1,000) for 2 hr at room temperature. After that, the nucleus was dyed by using 0.5 *μ*g/mL 4′,6-diamidino-2-phenylindole (with PBS) for 8 min. Finally, images were collected with a confocal microscope (SP8 DLS, Leica, Germany), analyzed by Imaris Viewer (Oxford Instruments, UK), and quantized by Image J (National Institutes of Health, USA). All the above operations were under dim light.

#### 2.4.5. qRT-PCR Analysis

Detailed steps of qRT-PCR analysis were performed in our previous study [27]. Briefly, total RNA was extracted using TRIzol reagent according to instructions (Invitrogen, Carlsbad, USA), and cDNA was synthesized using a cDNA synthesis kit (TransGen Biotech, AE311-03). Real-time quantitative PCR was performed with SYBR Green I Master Mix (Roche, Germany) on a Light-Cycler 480 system (Roche). *rpl7* was used as a housekeeping gene, and the primers in this study were listed in Table 2.

### 2.5. Statistical Analysis

All data were analyzed using GraphPad Prism 9 (GraphPad Software, Inc., La Jolla, USA). Data were evaluated by one-way ANOVA analysis after normality and homogeneity of variance were confirmed, and then Tukey's multiple comparisons were used to detect its significance. Polynomial contrasts were further used to determine linear, quadratic, and cubic effects of in vivo data according to Siringi et al. [28], then the equation with the higher *R*^2^ value was selected and presented in the figures. Comparisons were made between the two groups using the student test. The *P* < 0.05 was considered statistically significant. All data were shown as the means ± SEM (standard error of the mean).

## 3. Results

### 3.1. Growth Performance

The growth performance is shown in Table 3. In this study, final body weight, weight gain, specific growth rate, final fork length, and condition factor were not statistically significant among the different groups.

### 3.2. Excess Carbohydrates Activated Glycolysis and Suppressed Gluconeogenesis in Zebrafish and ZFL

**Figure 1:**
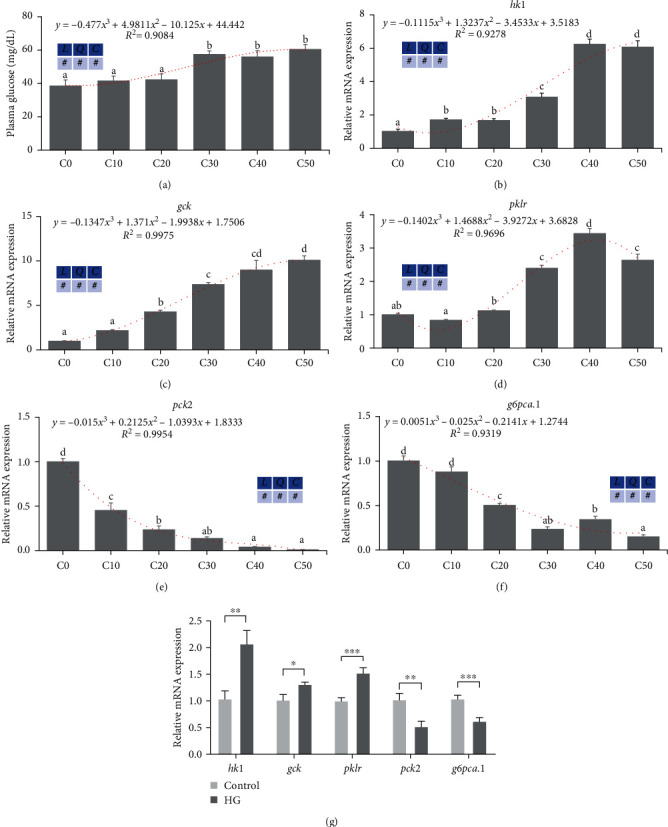
Excess carbohydrates activated glycolysis and suppressed gluconeogenesis in zebrafish and ZFL (means ± SEM, *n* = 6). C0, C10, C20, C30, C40, and C50 represent diets in which dextrin levels are 0%, 10%, 20%, 30%, 40%, and 50%, respectively. *L* represents a linear trend, *Q* represents a quadratic trend, *C* represents a cubic trend, and # represents the significant of the corresponding regressions. HG represents high glucose. (a) Plasma glucose, (b–d) glycolysis-related genes' expression in zebrafish liver, (e and f) gluconeogenesis-related genes' expression in zebrafish liver, and (g) glycolysis and gluconeogenesis related genes' expression in ZFL. Labeled means without a common letter differ among C0, C10, C20, C30, C40, and C50, *P* < 0.05 (one-way ANOVA, Duncan's post hoc test).  ^*∗*^*P* < 0.05,  ^*∗*^ ^*∗*^*P* < 0.01,  ^*∗*^ ^*∗*^ ^*∗*^*P* < 0.005 (two-tailed independent *t*-test).

The results showed that C30–C50 groups significantly increased plasma glucose content compared to other groups (*P* < 0.05) (Figure 1(a)). Meanwhile, high dietary dextrin levels (C30–C50 groups) significantly enhanced the expression level of liver glycolysis-related genes (*hk1*, *gck*, and *pklr*) (Figure 1(b)–1(d)), while suppressing the expression level of liver gluconeogenesis-related genes (*pck2* and *g6pc1a.1*) (Figures 1(e) and 1(f)) compared to other groups (*P* < 0.05). In addition, cubic changes in glucose content (Figure 1(a)), *hk1* (Figure 1(b)), *gck* (Figure 1(c)), and *pklr* (Figure 1(d)) were significant (*P* < 0.05), where *pklr* peaked in group C40. These glucose metabolism-related genes also significantly increased (*hk1*, *gck*, and *pklr*) or suppressed (*pck2* and *g6pca.1*) when incubated with high glucose in ZFL (*P* < 0.05) (Figure 1(g)).

### 3.3. Excess Carbohydrates Increased Lipid Accumulation by Promoting Lipogenesis in the Liver of Zebrafish

**Figure 2:**
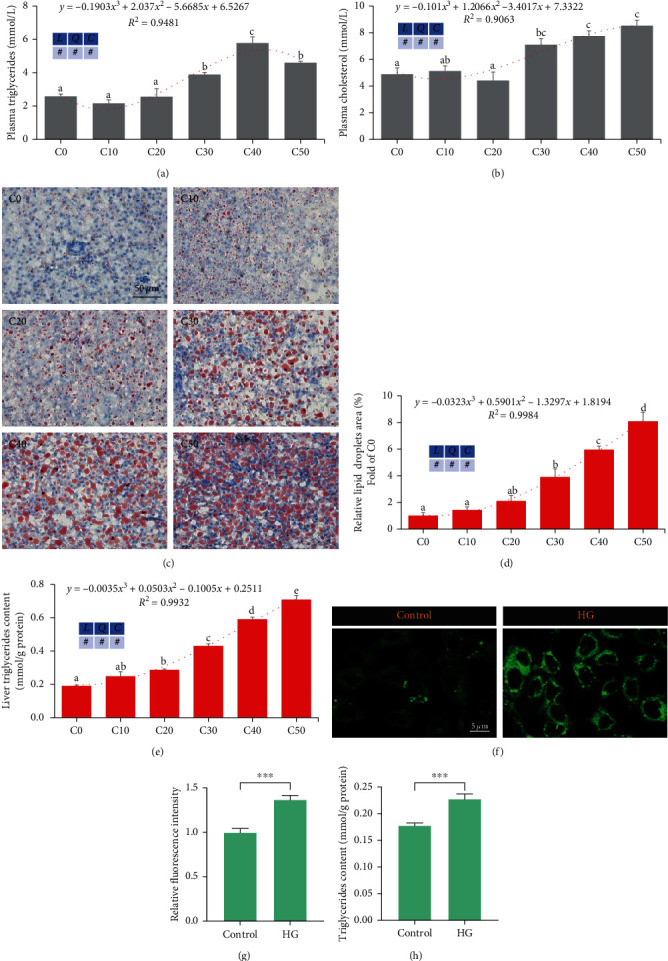
Excess carbohydrates elevated lipid accumulation in zebrafish and ZFL (means ± SEM, *n* = 6). C0, C10, C20, C30, C40, and C50 represent diets in which dextrin levels are 0%, 10%, 20%, 30%, 40%, and 50%, respectively. *L* represents a linear trend, *Q* represents a quadratic trend, *C* represents a cubic trend, and # represents the significant of the corresponding regressions. HG represents high glucose. (a) Plasma triglycerides, (b) plasma cholesterol, (c) representative images of the liver oil red O staining, scale bar, 50 *μ*m, (d) relative areas of lipid droplets in oil red O staining, (e) triglycerides in the liver of zebrafish, (f) representative images of BODIPY staining in ZFL, scale bar, 5 *μ*m, (g) lipid content was quantified by mean green fluorescence intensity by BODIPY 493/503 staining, and (h) triglycerides content in ZFL. Labeled means without a common letter differ among C0, C10, C20, C30, C40, and C50, *P* < 0.05 (one-way ANOVA, Duncan's post hoc test).  ^*∗*^ ^*∗*^ ^*∗*^*P* < 0.005 (two-tailed independent *t*-test).

As shown in Figure 2, with the increase of dietary dextrin levels, plasma triglycerides (Figure 2(a)) and cholesterol (Figure 2(b)) increased cubic (*P* < 0.05), where plasma triglycerides peaked in the C40 group. Liver lipid droplets (Figures 2(c) and 2(d)) and liver triglyceride content (Figure 2(e)) were also increased cubic (*P* < 0.05). In ZFL, high glucose significantly increased the intensity of the BODIPY signal (Figure 2(f)–2(g)) and triglyceride content compared to the control group (*P* < 0.05) (Figure 2(h)).

**Figure 3:**
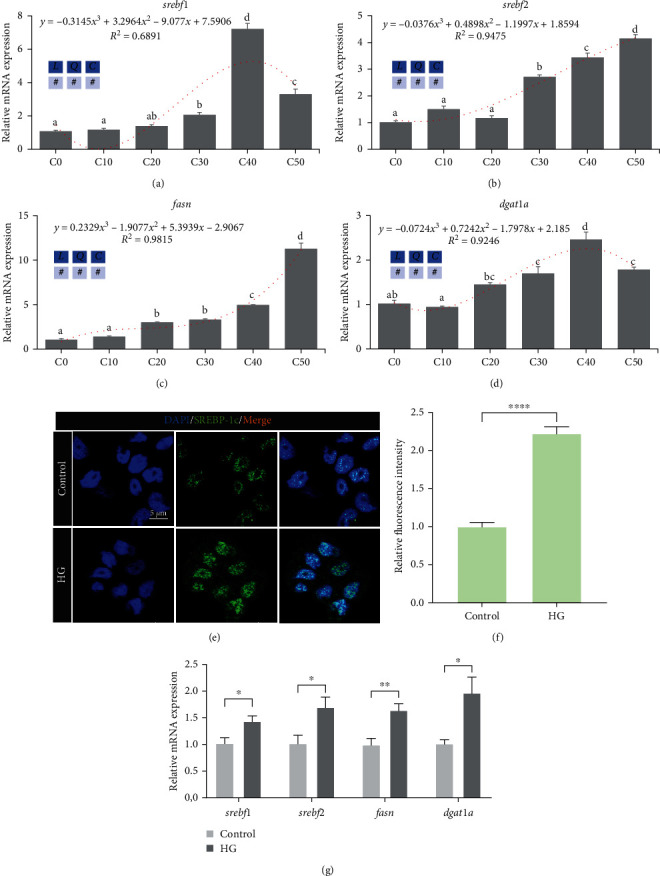
Excess carbohydrates promoted lipogenesis in zebrafish and ZFL (means ± SEM, *n* = 6). C0, C10, C20, C30, C40, and C50 represent diets in which dextrin levels are 0%, 10%, 20%, 30%, 40%, and 50%, respectively. *L* represents a linear trend, *Q* represents a quadratic trend, *C* represents a cubic trend, and # represents the significant of the corresponding regressions. HG represents high glucose. (a–d) Lipogenesis-related genes' expression in zebrafish liver, (e) representative images of SREBP-1c immunofluorescence staining in ZFL, scale bar, 5 *μ*m, (f) SREBP-1c was quantified by mean green fluorescence intensity by immunofluorescence staining in ZFL, and (g) lipogenesis-related genes' expression in ZFL. Labeled means without a common letter differ among C0, C10, C20, C30, C40, and C50, *P* < 0.05 (one-way ANOVA, Duncan's post hoc test).  ^*∗*^*P* < 0.05,  ^*∗*^ ^*∗*^*P* < 0.01,  ^*∗*^ ^*∗*^ ^*∗*^ ^*∗*^*P* < 0.005 (two-tailed independent *t*-test).

As shown in Figure 3, with the increase of dietary dextrin level, the relative expressions of hepatic lipogenesis genes (*srebf1*, *srebf2*, *fasn*, and *dgat1a*) (Figure 3(a)–3(d)) were cubic upregulated (*P* < 0.05), where *srebf1* and *dgat1a* with a peak at C40 group. In ZFL, we used immunofluorescence to assess the activation of SREBP-1c, an important transcription factor for glucose to lipid conversion, and found that high glucose induced an increase in the fluorescence intensity of SREBP-1c (Figures 3(e) and 3(f)). Consistent with the in vivo study, the expression level of ZFL lipogenesis genes (*srebf1*, *srebf2*, *fasn*, and *dgat1a*) also significantly increased when incubated with high glucose compared to the control group (*P* < 0.05) (Figure 3(g)).

### 3.4. Excess Carbohydrates Damaged the Liver Health of Zebrafish and Increased Inflammation-Related Genes' Expression in Zebrafish Liver and ZFL

**Figure 4:**
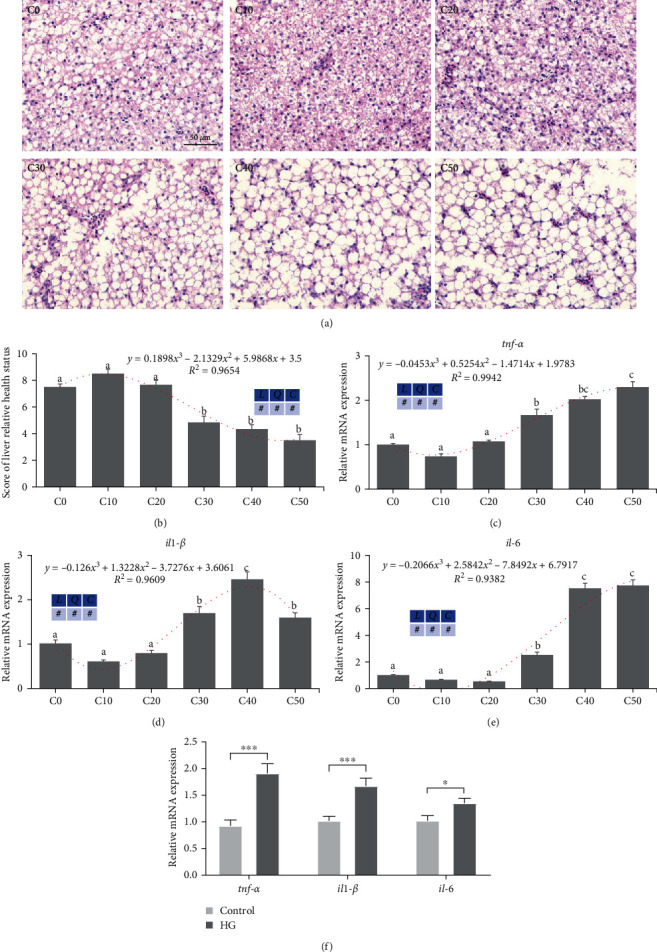
Excess carbohydrates damaged zebrafish liver health and increased inflammation-related genes in zebrafish liver and ZFL (means ± SEM, *n* = 6). C0, C10, C20, C30, C40, and C50 represent diets in which dextrin levels are 0%, 10%, 20%, 30%, 40%, and 50%, respectively. *L* represents a linear trend, *Q* represents a quadratic trend, *C* represents a cubic trend, and # represents the significant of the corresponding regressions. HG represents high glucose. (a) Representative images of the liver H&E staining, scale bar, 50 *μ*m, (b) the relative health status of the liver was scored according to the degree of vacuolization (1–2: severe damage, 3–4: moderate, 5–6: mild, 7–8: slight, 9–10: normal), (c–e) inflammation-related genes' expression in zebrafish liver, and (f) inflammation-related genes' expression in ZFL. Labeled means without a common letter differ among C0, C10, C20, C30, C40, and C50, *P* < 0.05 (one-way ANOVA, Duncan's post hoc test).  ^*∗*^*P* < 0.05,  ^*∗*^ ^*∗*^ ^*∗*^*P* < 0.005 (two-tailed independent *t*-test).

As shown in Figures 4(a) and 4(b), H&E staining of liver tissues in zebrafish showed no significant difference between C0–C20 groups. However, hepatocytes in the C30–C50 groups became hypertrophic and the nuclei disappeared, indicating liver damage in zebrafish. As demonstrated in Figure 4(c)–4(e), zebrafish showed no significant alteration in liver inflammation-related genes (*tnf-α*, *il1-β*, and *il-6*) across the C0–C20 groups. However, as the dietary dextrin levels increased, a cubic upregulation was observed in the hepatic inflammation genes (*tnf-α*, *il1-β*, and *il-6*) (*P* < 0.05). These molecular indicators (*tnf-α*, *il1-β*, and *il-6*) also significantly increased when incubated with high glucose in ZFL compared to the control group (*P* < 0.05) (Figure 4(f)).

## 4. Discussion

In this study, we evaluated carbohydrate-induced responses by using dextrin as a carbohydrate source in zebrafish. However, the present results showed that different dietary dextrin levels had no significant effect on the growth performance of zebrafish. Muscle growth in fish is not linear, it occurs through a combination of the recruitment of new muscle fibers (hyperplasia) and an increase in the size of existing fibers (hypertrophy) in postjuvenile stages [29–31]. Unlike other fish, the number of muscle fibers in zebrafish is determined and fixed at birth, and growth is solely due to the hypertrophy of fibers already formed [32]. These conditions prevent normal muscle growth, and zebrafish only grow to the appropriate ultimate body size (3–5 cm) [29, 30]. In our study, the ultimate fork length (∼3.6 cm) did not exhibit a significant variance when subjected to varying diets, implying that zebrafish may have achieved their appropriate body proportions.

Blood glucose concentration is an important indicator of glucose homeostasis in aquatic animals [2]. In the present study, zebrafish fed diets of 30%–50% dextrin significantly increased plasma glucose levels, which is consistent with our previous study, a 40% dextrin diet caused postprandial hyperglycemia in adult zebrafish [33]. In some aquatic animals, blood glucose was directly proportional to dietary carbohydrate levels, such as abalone (*Haliotis discus hannai*) [2], mud crab (*Scylla paramamosain*) [34], and cobia (*Rachycentron canadum*) [35]. Various studies have shown that glycolysis-related genes are induced by high carbohydrate levels [36, 37]. The liver plays a key role in regulating glycolipid metabolism and controlling many processes [38], including the glycolysis pathway. Hexokinase, glucokinase (an isoenzyme of hexokinase), and pyruvate kinase are key regulatory enzymes in the glycolysis pathway, which is a necessary common stage for glucose catabolism in aquatic animals [34, 39]. Previous studies have shown that hexokinase activity may be significantly induced by dietary carbohydrates [39]. In grass carp (*Ctenopharyngodon idella*), high dietary carbohydrates (50% of maize starch) significantly increased liver glucokinase and pyruvate kinase activity and gene expression [7]. In blunt snout bream (*Megalobrama amblycephala*), fish fed the dextrin diet (33%) showed the highest liver glucokinase and pyruvate kinase compared to other carbohydrate sources [40]. In the present study, liver gene expression of *hk1*, *gck*, and *pklr* was significantly elevated in 30%–50% dietary dextrin groups compared to 0% dietary dextrin group, indicating that increased dietary carbohydrate content activated the glycolysis pathway, which is similar to the results of zebrafish fed with wheat starch as carbohydrate [17] and gibel carp fed with corn starch as carbohydrate [41]. Generally, elevated plasma glucose levels to high carbohydrate consumption may enhance glycolysis coupled with suppression of gluconeogenesis [42]. In the present study, two key genes involved in the gluconeogenesis pathway are significantly decreased in the liver of zebrafish when fed excess dietary carbohydrates (30%–50% dextrin), and this result is similar to that in grass carp [43] and gibel carp [41]. These in vivo results are consistent with the results in ZFL when incubated in high glucose. Our results were supported by a recent study showing that high glucose activated glycolysis and suppressed gluconeogenesis in ZFL which is a suitable fish cell line for studying glucose metabolism [44]. Therefore, using zebrafish as a model can promote the study of glucose metabolism and provide basic data for a better understanding of the physiological mechanism in fish.

Abnormal glycolipid metabolism is closely related to hypertriglyceridemia, obesity, fatty liver disease, and diabetes [38, 45]. Numerous studies have reported that excessive dietary carbohydrate intake leads to glycolipid metabolism disorder, which affects health, retarded growth, and ultimately causes death of aquatic animals [4, 46–48]. In the present study, plasma triglycerides and cholesterol were significantly increased when fed 30%–50% of dietary dextrin compared to the other groups, which may indicate that too much carbohydrate causes lipid metabolism disorder in zebrafish. In this study, the oil red O staining and triglyceride content in the liver were significantly increased when fed to 30%–50% of the dietary dextrin groups compared to 0% of the dietary dextrin group. Evidence suggests that high level of lipogenesis of triglyceride is an important abnormal signal in nonalcoholic fatty liver disease (NAFLD) and may be a key event leading to massive steatosis [49]. The induction of lipogenesis is primarily controlled by SREBP-1c, which directly activates the expression of more than 30 genes dedicated to fatty acid uptake and triglyceride synthesis [50, 51]. In the present study, high glucose significantly increased the SREBP-1c expression in ZFL. And the triglyceride content in ZFL was also significantly increased when incubated in high glucose. Overactivation of SREBP-1c would lead to triglyceride accumulation and lead to hepatic steatosis [52]. Elevated SREBP-1c was found in patients with histologically diagnosed NAFLD [53]. In zebrafish, copper could induce triglyceride accumulation through the SREBP-1 pathway, accompanied with lipogeneses-related genes' expression such as *fas* and *dgat1a* [54]. Similarly, in the present study, lipogenic genes (*srebf1*, *srebf2*, *fasn*, and *dgat1a*) were significantly increased when fed 30%–50% of the dietary dextrin groups compared to 0% of the dietary dextrin group. These genes have similar changes when incubated ZFL in a high glucose medium. These results indicated that zebrafish may be a good model for a better understanding of the underlying mechanism from carbohydrate to lipid conversion.

Histological changes in the liver are crucial for understanding nutritional-related pathological alterations in fish [55]. Excessive dietary carbohydrate intake would alter liver histological status, and cause the disappearance of the nuclear membrane in cells, karyopyknosis, and hepatocyte necrosis [27, 56]. In the present study, 30%–50% of dietary dextrin resulted in severe vacuolation in the liver of zebrafish. Previous studies have demonstrated that *tnf-α*, *il1-β*, and *il-6* are proinflammatory genes, which are considered to be molecular indicators when the organism under inflammation response [44, 57]. And numerous studies have shown that high-carbohydrate diet could induce the proinflammatory genes' expression in different aquatic animals, such as blunt snout bream (*M. amblycephala*) [58], Nile tilapia (*Oreochromis niloticus*) [47], Atlantic cod (*Gadus morhua* L.) [59]. Similarly, in the present study, 30%–50% of dietary dextrin significantly increased the liver gene (*tnf-α*, *il1-β*, and *il-6*) expression in zebrafish. Transcriptional levels of these genes were also significantly increased in ZFL when incubated with high glucose. The present results were further supported by a previous study in ZFL that high glucose induced the expression of *tnf-α*, *il1-β*, and *il-6* [44]. All of these indicated that high dietary carbohydrates damaged the liver health of zebrafish.

In the present study, cellulose was used to maintain balance with dextrin in experimental diets. Although cellulose cannot be digested by endogenous enzymes in fish, it may affect the structure and function of the digestive tract [60, 61]. In rainbow trout (*Salmo gairdnerii Richardson*), stomach : hind gut ratio was proportional to the ratio of dietary cellulose to protein level, but liver weight was not significantly influenced by diets [62]. Zebrafish fed the high-cellulose/low-protein (60% cellulose, 8.5% protein) diet showed significantly reduced gut mass, but increased gut length and surface area compared to the low-cellulose/high-protein (15% cellulose, 40.5% protein) diet [61]. In fish, digestive tract and liver tissues are important metabolic organs. In the present study, liver triglycerides and cholesterol contents were proportional to the ratio of dietary dextrin to cellulose. The results indicated that high-cellulose/low-dextrin diets led to reduced absorption and conversion of nutrients, such as lipid. In addition, interestingly, some present results of liver gene expressions (*pklr*, *srebf1*, *dgat1a*, and *il1-β*) have peak values in C40 group (10% cellulose, 40% dextrin) but not in C50 group (0% cellulose, 50% dextrin), which indicated the physiological status of fish could be significantly adjusted by low dietary cellulose.

## 5. Conclusion

The current investigation evaluated the feasibility of the zebrafish model to study glucose metabolism in fish both in vivo (zebrafish) and in vitro (ZFL). In the in vivo study, we investigated the response of zebrafish to different dietary dextrin levels and found that 0%–20% dietary dextrin had no significant effect on glucolipid metabolism and liver health, while excess dietary dextrin (30%–50%) activated glycolysis, suppressed gluconeogenesis, elevated lipid accumulation by promoting lipogenesis, and damaged liver health. In the in vitro study, we incubated ZFL to high glucose medium (20 mM) and found that high glucose also activated glycolysis and suppressed gluconeogenesis, elevated lipid accumulation by promoting lipogenesis, and increased inflammation levels. All the present results indicate that zebrafish is an optimal model for studying glucose metabolism in fish. Therefore, we recommend 20% dietary dextrin as a control group and 40% dietary dextrin as a high-carbohydrate treatment for adult zebrafish in a future study.

## Figures and Tables

**Table 1:** Formulation and proximate composition (g/kg on a dry weight basis) of the experimental diets.

Ingredients	Diets (g/kg)^a^					
	C0	C10	C20	C30	C40	C50
Casein^b^	380.0	380.0	380.0	380.0	380.0	380.0
Gelatin^c^	8.0	8.0	8.0	8.0	8.0	8.0
Soybean oil^d^	50.0	50.0	50.0	50.0	50.0	50.0
Dextrin^e^	0.0	100.0	200.0	300.0	400.0	500.0
Cellulose^f^	500.0	400.0	300.0	200.0	100.0	0.0
Mineral premix^g^	10.0	10.0	10.0	10.0	10.0	10.0
Choline chloride^h^	3.0	3.0	3.0	3.0	3.0	3.0
Vitamin premix^i^	7.0	7.0	7.0	7.0	7.0	7.0
Monocalcium phosphate^j^	30.0	30.0	30.0	30.0	30.0	30.0
L-Threonine^k^	1.5	1.5	1.5	1.5	1.5	1.5
L-Arginine^l^	8.5	8.5	8.5	8.5	8.5	8.5
L-Tryptophan^m^	2.0	2.0	2.0	2.0	2.0	2.0
Dextrin/cellulose	0.0	0.25	0.67	1.5	4.0	–
Proximate composition (g/kg)						
Moisture	118.0	120.5	114.3	121.4	115.6	117.8
Crude protein	375.6	379.5	378.1	374.4	372.0	372.5
Crude lipid	43.7	49.0	46.3	45.5	45.2	44.7
Crude ash	33.5	33.3	33.5	32.8	31.9	31.3
Fiber	424.8	349.6	275.2	201.6	120.7	3.9
Gross energy (MJ/kg diet)	18.8	19.4	19.7	20.3	20.7	21.0
Carbohydrate/fiber	0.01	0.19	0.55	1.11	2.61	110.21
Crude protein/gross energy	20.0	19.5	19.2	18.4	18.0	17.8

*Note*. ^a^C0, C10, C20, C30, C40, and C50 represent diets in which dextrin levels of 0%, 10%, 20%, 30%, 40%, and 50%, respectively. ^b^Casein: purchased from Sigma (Product number: C3400). ^c^Gelatin: from Sinopharm Chemical Reagent Co., Ltd., China. ^d^Soybean oil: from Yihai Kerry Arawana Holdings Co., Ltd., Shanghai, China. ^e^Dextrin: from Sinopharm Chemical Reagent Co., Ltd., China. ^f^Microcrystalline cellulose: from Shandong Liujia Pharmaceutical Excipients Co., Ltd., Jining, Shandong, China. ^g^Mineral premix (mg/kg diet): CoCO_3_, 0.65; CuSO_4_·5H_2_O, 9.00; FeSO_4_·7H_2_O, 8.34; NaCl, 400.00; MgO, 240.00; MnSO_4_·H_2_O, 22.85; KI, 0.50; Na_2_SeO_3_, 0.01; CaCO_3_, 1,860.00; ZnSO_4_·7H_2_O, 14.30; microcrystalline cellulose, 7,444.35. ^h^Choline chloride: from Guangdong Nutriera Group, Guangzhou, China. ^i^Vitamin premix (mg/kg diet): tocopherol acetate, 100; sodium menadione bisulfate, 25; retinyl acetate, 6.9; cholecalciferol, 0.05; thiamin, 30; riboflavin, 30; pyridoxine, 20; cyanocobalamin, 0.1; nicotinic acid, 200; folic acid, 15; ascorbic acid, 1,000; inositol, 500; biotin, 3; calcium pantothenate, 100; microcrystalline cellulose 4,669.95. ^j^Monocalcium phosphate: from Sinopharm Chemical Reagent Co., Ltd., China. ^k^L-Threonine: purchased from Aladdin (CAS number: 72-19-5). ^l^L-Arginine: purchased from Aladdin (CAS number: 74-79-3). ^m^L-Tryptophan: purchased from Aladdin (CAS number: 73-22-3).

**Table 2:** Sequences of the primers used for qRT-PCR.

Gene name	Forward (F) and reverse (R) primer (5′–3′)	Accession no.	Product length (bp)
Ribosomal protein L7 (*rpl7*)	F: CAGAGGTATCAATGGTGTCAGCCC	NM_213644.2	119
	R: TTCGGAGCATGTTGATGGAGGC		

Hexokinase 1 (*hk1*)	F: CTTGGGTGTAGAGCCGTCTG	NM_213252.1	142
	R: ACGTGGGGTGTTCTTGTTGT		

Glucokinase (*gck*)	F: CACCGCTGACCTGCTATGAT	NM_001045385.2	102
	R: AGTCGGCCACTTCACATACG		

Pyruvate kinase L/R (*pklr*)	F: TCCTGGAGCATCTGTGTCTG	NM_201289.1	144
	R: GTCTGGCGATGTTCATTCTT		

Phosphoenolpyruvate carboxykinase 2 (*pck2*)	F: TGCCTGGATGAAATTTGACA	NM_213192.1	106
	R: GGCATGAGGGTTGGTTTTTA		

Glucose-6-phosphatase catalytic subunit 1a, tandem duplicate 1 (*g6pca.1*)	F: GCTCATTTCCCACACCAAGT	NM_001316336.1	150
	R: ATAAAAGCCCACAGCGAATG		

Sterol regulatory element binding transcription factor 1 (*srebf1*)	F: ATGGCGGAAGACAGCAAG	NM_001105129.1	107
	R: AGCGGGTTAAAGGACAGAA		

Sterol regulatory element binding transcription factor 2 (*srebf2*)	F: CACACTCTTCTCTCTGCCCG	NM_001089466.1	165
	R: GATGTCGGTGAGTGAAGGGG		

Fatty acid synthase (*fasn*)	F: GGAGCAGGCTGCCTCTGTGC	XM_009306807.3	128
	R: TTGCGGCCTGTCCCACTCCT		

Diacylglycerol O-acyltransferase 1a (*dgat1a*)	F: CCAAAGCTCGAACCCTGTCT	NM_199730.1	104
	R: GTGTGTGAGGTTTCCCGGAT		

Tumor necrosis factor a (*tnf-α*)	F: GGGCAATCAACAAGATGGAAG	NM_212859.2	250
	R: GCAGCTGATGTGCAAAGACAC		

Interleukin 1, beta (*il1-β*)	F: CGCCCTGAACAGAATGAAGCAC	NM_212844.2	128
	R: AAGACGGCACTGAATCCACCAC		

Interleukin 6 (*il-6*)	F: CACGGAAAGATGTCTAACGCGAAT	NM_001261449.1	141
	R: TTTATGGCCTCCAGCAGTCGTTT		

**Table 3:** Growth performance of male zebrafish fed different experimental diets for 6 weeks.

Parameters	Diets^a^						Contrast		
	C0	C10	C20	C30	C40	C50	Linear	Quadratic	Cubic
IBW (mg)	250.4 ± 2.0	250.7 ± 1.6	250.6 ± 1.2	250.7 ± 0.8	250.4 ± 1.8	250.6 ± 1.7	ns	ns	ns
FBW (mg)	392.4 ± 6.3	403.9 ± 5.3	392.2 ± 11.1	390.2 ± 11.9	388.5 ± 7.1	384.6 ± 2.6	ns	ns	ns
WG (%)	56.7 ± 1.8	61.1 ± 1.4	56.6 ± 4.6	55.6 ± 5.0	55.2 ± 3.3	53.5 ± 1.4	ns	ns	ns
SGR (%/day)	1.1 ± 0.0	1.1 ± 0.0	1.1 ± 0.1	1.1 ± 0.1	1.0 ± 0.0	1.0 ± 0.0	ns	ns	ns
IFL (mm)	28.6 ± 0.3	28.3 ± 0.3	29.6 ± 0.1	27.5 ± 1.1	28.9 ± 0.5	28.3 ± 0.6	ns	ns	ns
FFL (mm)	36.2 ± 0.1	36.2 ± 0.3	36.8 ± 0.2	36.4 ± 0.4	36.5 ± 0.4	36.2 ± 0.2	ns	ns	ns
CF (%)	0.83 ± 0.02	0.85 ± 0.03	0.79 ± 0.03	0.81 ± 0.02	0.80 ± 0.02	0.81 ± 0.01	ns	ns	ns

*Note*. Data in the table are represented as means ± SEM (*n* = 3). ns, nonsignificant; IBW, initial body weight; FBW, final body weight; WG, Weight gain = 100 × (Final body weight − Initial body weight)/Initial body weight; IFL, initial fork length; FFL, final fork length; SGR, Specific growth rate = 100 × [ln (Final body weight) – ln (Initial body weight)]/days; CF, Condition factor = 100 × (Body weight/Body length^3^). ^a^C0, C10, C20, C30, C40, and C50 represent diets in which dextrin levels of 0%, 10%, 20%, 30%, 40%, and 50%, respectively.

## Data Availability

The raw data from the present study can be obtained upon request to the corresponding author who was the project leader of the present work.
